# Immunogenicity of BNT162b2, BBIBP-CorV and Gam-COVID-Vac vaccines and immunity after natural SARS-CoV-2 infection—A comparative study from Novi Sad, Serbia

**DOI:** 10.1371/journal.pone.0263468

**Published:** 2022-02-02

**Authors:** Vladimir Petrović, Vladimir Vuković, Aleksandra Patić, Miloš Marković, Mioljub Ristić

**Affiliations:** 1 Faculty of Medicine, University of Novi Sad, Novi Sad, Serbia; 2 Institute of Public Health of Vojvodina, Novi Sad, Serbia; 3 Department of Immunology, Faculty of Medicine, Institute of Microbiology and Immunology, University of Belgrade, Belgrade, Serbia; University of Pittsburgh, UNITED STATES

## Abstract

**Background:**

Mass vaccination is the key element in controlling current COVID-19 pandemic. Studies comparing immunogenicity of different COVID-19 vaccines are largely lacking. We aimed at measuring anti-S antibody (Ab) levels in individuals fully vaccinated with BNT162b2, BBIBP-CorV and Gam-COVID-Vac, as well as in COVID-19 convalescents.

**Methods:**

In this cross-sectional study, serum was collected from 400 age- and sex-matched participants, 100 fully vaccinated with BNT162b2, 100 with BBIBP-CorV and 100 with Gam-COVID-Vac on the 28^th^ day after the second vaccine dose, and 100 recovered from COVID-19 at least 28 days after symptom(s) resolution. Sera were analyzed using the LIAISON SARS-CoV-2 S1/S2 IgG assay (DiaSorin, Saluggia, Italy). Wilcoxon rank-sum or Kruskal–Wallis tests was used for comparison of Ab levels.

**Results:**

Highest mean value (210.11, SD = 100.42) was measured in the BNT162b2 group, followed by Gam-COVID-Vac (171.11, SD = 120.69) and BBIBP-CorV (68.50, SD = 72.78) AU/mL (p<0.001). Significant differences in antibody levels were found between BNT162b2 and BBIBP-CorV (p<0.001), BNT162b2 and Gam-COVID-Vac (p = 0.001), as well as BBIBP-CorV and Gam-COVID-Vac groups (p<0.001). Percentage of seropositive was 81% in the convalescent group, 83% in BBIBP-CorV vaccinated and 100% in BNT162b2 and Gam-COVID-Vac. When comparing measured antibody levels in vaccinated to those in COVID-19 recovered, significantly higher antibody levels were found for vaccinated with BNT162b2 (p<0.001), and with Gam-COVID-Vac (p<0.001), while for BBIBP-CorV there was no statistically significant difference (p = 0.641).

**Conclusions:**

All three investigated vaccines, BNT162b2, BBIBP-CorV and Gam-COVID-Vac, provide robust immune response 28 days after the second dose of vaccine, in the majority of participants. All individuals vaccinated with BNT162b2 and Gam-COVID-Vac seroconverted, while in vaccinated with BBIBP-CorV and COVID-19 recovered seroconversion rates were lower. Although less potent compared to other two vaccines, immune response after BBIBP-CorV was similar to response measured in convalescents. Challenge still remains to examine dynamics and durability of immunoprotection.

## Introduction

Mass vaccination is the key element in controlling the coronavirus disease 2019 (COVID-19) pandemic and it is widely acknowledged that implementation of global vaccination programme is pre-requisite for world to return to normality [1, 2]. Joint efforts to put the SARS-CoV-2 epidemic under control became a global priority and has resulted in prompt action in development of the vaccines [3]. Various vaccine platforms, including mRNA, adenovirus vectors, and inactivated virus, have been used for the SARS-CoV-2 vaccine development [4]. Data from phase 3 clinical trials of different vaccines showed encouraging efficacies against symptomatic COVID-19 ranging from 67% to as much as 95% [5].

In Serbia, the COVID-19 vaccination campaign started on December 24, 2020. Recommended immunization programme was implemented and vaccines were offered free of charge. Serbian Medicines Agency approved four vaccines for use in population, namely Pfizer-BioNTech BNT162b2 (Comirnaty^®^) was approved on December 23^rd^, 2020, Sinopharm BBIBP-CorV (Vero Cell^®^) on January 3^rd^, 2021, Gam-COVID-Vac (Sputnik V^®^) on January 18^th^, 2021, and Oxford/AstraZeneca ChAdOx1-S/nCoV-19 AZD1222 (Vaxzevria^®^) on February 20^th^, 2021 [6]. These vaccines are based on different platforms and have diverse mechanisms of action, which were described elsewhere [7–9].

It is generally accepted that higher antibody (Ab) levels and neutralizing antibodies specific for spike (S) protein of SARS-CoV-2 levels in particular, are likely to protect against the disease [10]. Though all vaccines against SARS-CoV-2 have been shown to induce good humoral immune response, including neutralizing antibodies against the S protein [4], studies comparing immunogenicity of diverse COVID-19 vaccines are largely lacking. Thus, measuring anti-S Ab levels in circulation of similar individuals vaccinated with different vaccines, at the same time points following vaccination and using the same immunological assay that quantifies antibodies binding to S protein, could enable a comparison of the immunogenicity between different vaccines. On the other hand, natural immunity after infection may differ from post-vaccination immunity. Anti-S antibodies are detected in many but not all convalescent individuals, with levels varying much between individuals. Moreover, both neutralizing antibody titers and total anti-S antibody titers have positively correlated with COVID-19 disease severity [4, 10].

In the present study we analyzed and compared humoral immune response and assessed anti-S antibody levels in individuals fully vaccinated with one of the three different COVID-19 vaccines (BNT162b2, BBIBP-CorV and Gam-COVID-Vac) as well as in the COVID-19 convalescents.

## Material and methods

### Study cohort

Healthy participants, aged 18 years and older, from the City of Novi Sad (main administrative centre of the Autonomous Province of Vojvodina, Serbia) were recruited. Individuals without evidence of prior SARS-CoV-2 infection who were vaccinated with two doses of vaccine, 21 days apart, in the period between January and April 2021, were enrolled. Three groups of participants were selected from the database for COVID-19 immunization monitoring (100 individuals vaccinated with BNT162b2, 100 with BBIBP-CorV and 100 with Gam-COVID-Vac) and matched by sex- and 10-year age categories. After recruitment of vaccinated participants, we also searched for matched COVID-19 convalescents based on the age- and sex- distribution. Potentially eligible participants were interviewed by phone and were scheduled for blood sample collection for anti-S SARS-CoV-2 antibodies assessment on the 28^th^ day after receiving the second vaccine dose. Exclusion criteria for vaccinated participants were: previous diagnosis of COVID-19 or its laboratory confirmation (by reverse transcription polymerase chain reaction—RT-PCR or rapid diagnostic test for detection of SARS-CoV-2 antigen—RDT-Ag) [11], and presence of any signs or symptoms related to SARS-CoV-2 infection 10 days before vaccination, in the period between two doses, or during 28 days after the second dose of vaccine. In order to enable comparison of the Ab responses after immunization with three different vaccines with those after natural infection, we also enrolled 100 COVID-19 convalescent individuals. All of them had laboratory-confirmation of the prior SARS-CoV-2 infection obtained by either RT-PCR or RTD-Ag and have not been previously vaccinated against COVID-19. Clinical presentation of COVID-19 disease was defined as asymptomatic, if no symptoms were present during the course of PCR or RTD-Ag positivity; mild, if patients experienced symptoms without clinical or radiological signs of pneumonia; severe, in case COVID-19 pneumonia was confirmed, and oxygenation was required; and critical if patients with pneumonia required invasive mechanical ventilation [12]. Among 100 blood samples collected, 10 were from asymptomatic patients, 70 from mild, 19 from severe and one sample from critically-ill patient, respecting sex- and age- distribution in each group.

### Vaccines

We analyzed antibody levels after vaccination with Pfizer-BioNTech BNT162b2 (Comirnaty^®^), Sinopharm BBIBP-CorV (Vero Cell^®^) and Gam-COVID-Vac (Sputnik V^®^), that have similar immunization schedule of three weeks between two doses. We did not analyze antibody levels after Oxford/AstraZeneca ChAdOx1-S/nCoV-19 AZD1222 (Vaxzevria^®^) due to a longer interval of twelve weeks between the two doses.

### Blood collection and measurement of SARS-CoV-2 IgG antibody levels against spike protein

All blood samples were collected and analyzed in the Centre for Virology of the Institute of Public Health of Vojvodina, Novi Sad. Blood samples were collected 28 days after the second dose of vaccine or at least 28 days after symptom resolution in convalescents. Samples were taken in collection tubes, centrifuged and serum was separated from the clot. Serum samples were tested with assay designed to measure total amount of antibodies against the S protein, which is the principal target for neutralizing antibodies. IgG antibody levels against S1 and S2 subunit of spike protein were measured in each sample using the quantitative LIAISON SARS-CoV-2 S1/S2 IgG assay (DiaSorin, Saluggia, Italy) which is able to detect and quantify antibodies with high sensitivity and specificity using the indirect chemiluminescence immunoassay (CLIA) method performed on the LIAISON^®^ XL Analyzer. Specific recombinant S1 and S2 antigens were used to coat magnetic particles (solid phase). Anti S1/S2 antibodies from the sample bound to particles are detected by mouse monoclonal antibodies against human IgG that are linked to an isoluminol derivative (isoluminol–antibody conjugate). Subsequently, the light signal, which indicates the amount of isoluminol–antibody conjugate, was measured using a photomultiplier. The obtained value, expressed in relative light units (RLU), represents the concentration of IgG to SARS-CoV-2 present in calibrators, samples, or controls. The analyzer automatically calculates concentrations of SARS-CoV-2 S1/S2 IgG antibody in arbitrary units (AU/mL) and grades the results. All samples with IgG titers below 12.0 AU/mL were considered negative. Values ≥12.0 AU/mL and <15.0 AU/mL were considered equivocal (unknown immunity status), while the samples with titers greater than or equal to 15.0 AU/mL were considered positive for IgG antibodies against the spike protein of SARS-CoV-2, as recommended by the manufacturer [13].

### Statistical analyses

Summary statistics are presented by each of the four study groups, stratified by sex, 10-year age categories, and for COVID-19 convalescent participants, by clinical presentation of disease. Categorical variables are presented as absolute frequencies and percentages (%) and continuous data as mean with standard deviation (SD) and median with interquartile range (IQR). To check for normality of the data, we used the Shapiro–Wilk/Kolmogorov–Smirnov test as well as visual inspection of the distribution plots. For comparison of antibody titers between groups, Wilcoxon rank-sum (Fisher’s exact test where appropriate) or Kruskal–Wallis test was used. In addition, Pearson’s correlation was used to explore potential correlations between antibody levels and different variables. Finally, we considered positive/negative result, based on the Ab cutoff value (15.0 AU/mL) as a dichotomous variable and performed comparison using Pearson’s Chi-square test or Fisher exact test, where appropriate. For the purpose of statistical analyses and graphical presentation of the data, any serologic value below the lower limit of quantitation (3.80 AU/mL) was set to 3.79 while those above the upper limit (>400 AU/mL) were set to 401. Visual presentation of the range of antibody levels across sex- and age-categories was done using the boxplots. All statistical analyses and graphical presentation of the data were performed using statistical software package Stata v.16 (College Station, TX: StataCorp LLC. 2019). Statistical significance was considered as an alpha level of 0.05, two-sided.

### Ethical considerations

The protocol was approved by the Ethics Committee of the Institute of Public Health of Vojvodina, Novi Sad under the number 01-860/1/2021. Oral informed consent was obtained from each participant, in accordance with national regulations. The Ethics Committee approved the use of oral consent to minimize contact time between the medical personal and the participants as the study was conducted during the pandemic. All data were anonymized before the authors accessed it and none of the authors of this study were involved in treatment of the patients included in the analysis.

## Results

A total of 400 serum samples from age- and sex-matched participants were analyzed: 300 samples were from the vaccinated participants evenly distributed between three investigated vaccines (100 samples in each group), while the remaining 100 samples were from the unvaccinated subjects who recovered from COVID-19. Mean age was 51.06 (SD = 12.17), 51.02 (SD = 12.02), 51.13 (SD = 11.93) years for participants vaccinated with BNT162b2, BBIBP-CorV and Gam-COVID-Vac, respectively, and 51.53 years (SD = 11.95) for COVID-19 recovered. In each of these groups there were 59% of female participants.

Noticable differences in antibody levels were observed in subjects that received different vaccines, as presented in Table 1.

**Table 1:** Antibody levels on the 28^th^ day from the administration of the second dose of vaccine or after COVID-19 recovery, stratified by sex, age and clinical presentation of COVID-19.

	Partici-pants (%)	BNT162b2 vaccine (n = 100)			BBIBP-CorV vaccine (n = 100)			Gam-COVID-Vac vaccine (n = 100)			COVID-19 recovered (n = 100)		
		mean (SD), AU/mL	median (IQR, 25–75), AU/mL	p-value^1^	mean (SD), AU/mL	median (IQR, 25–75), AU/mL	p-value^1^	mean (SD), AU/mL	median (IQR, 25–75), AU/mL	p-value^1^	mean (SD), AU/mL	median (IQR, 25–75), AU/mL	p-value^1^
**Total**	100	210.11 (100.42)	207 (133–280)	NA	68.5 (72.78)	47.8 (22.95–83.95)	NA	171.11 (120.69)	133.5 (78.25–241)	NA	81.23 (86.46)	46.1 (19.3–121.5)	NA
**Sex**													
Male	41	201.86 (102.12)	199 (121–243)	0.497	61.89 (58.38)	48.1 (15.2–78.6)	0.508	164.03 (114.31)	135 (72.9–228)	0.75	83.12 (67.81)	68.6 (21.1–126)	0.259
Female	59	215.85 (99.69)	208 (141–286)		73.09 (81.48)	47.5 (27.2–89.2)		176.03 (125.66)	129 (82.2–258)		79.92 (97.9)	42.7 (18.5–109)	
**Age category**													
20–29	2	192.5 (86.97)	192.5 (131–254)	0.132	112.45 (92.7)	112.45 (46.9–178)	0.179	363 (53.74)	363 (325–401)	0.379	48.8 (3.96)	48.8 (46–51.6)	<0.001
30–39	11	289.14 (103.48)	297 (213–401)		108.54 (123.34)	55.1 (34.6–118)		131.4 (62.43)	121 (74.3–168)		32.42 (38.65)	18.8 (14.9–30.6)	
40–49	38	207.01 (106.25)	183.5 (122–286)		49.91 (43.27)	41.9 (13.9–69.7)		167.57 (129.63)	127 (60.8–228		51.39 (58.49)	26.6 (13.3–70.7)	
50–59	30	213.77 (92.72)	209 (140–278)		78.77 (85.36)	50.35 (27.2–78.6)		194.5 (122.35)	162 (89.3–285)		106.06 (111.53)	66.15 (21.6–123)	
60–69	7	197.71 (80.46)	217 (121–239)		56.88 (60.77)	31.6 (15.1–93.7)		141.84 (127.41)	103 (38.1–195)		142.49 (117.76)	138 (44.3–170)	
70–79	11	145.59 (77.48)	143 (62.3–225)		69.95 (42.48)	63.4 (30.8–110)		148.89 (111.07)	122 (64.3–209)		135.45 (37.78)	132 (96.2–167)	
80+	1	181	181		3.79	3.79		106	106		47.3	47.3	
**COVID-19, clinical presentation**													
asymptomatic	10	∙∙	∙∙	NA	∙∙	∙∙	NA	∙∙	∙∙	NA	23.12 (27.3)	10.5 (6.98–24.7)	<0.001
mild	70	∙∙	∙∙		∙∙	∙∙		∙∙	∙∙		59.07 (50.04)	41.9 (20–93.5)	
severe	19	∙∙	∙∙		∙∙	∙∙		∙∙	∙∙		190.26 (120.86)	139 (109–301)	
critical	1	∙∙	∙∙		∙∙	∙∙		∙∙	∙∙		142	142	

**Note**: For statistical processing and presentation of data, results below the minimum detectable value of the assay (<3.8) was interpreted as 3.79, and above the maximum detectable value (> 400) as 401. NA = not applicable.

^1^Wilcoxon rank-sum (Fisher’s exact test where appropriate) or Kruskal–Wallis test; p-value referes to difference between variables within the same group.

**Fig 1:**
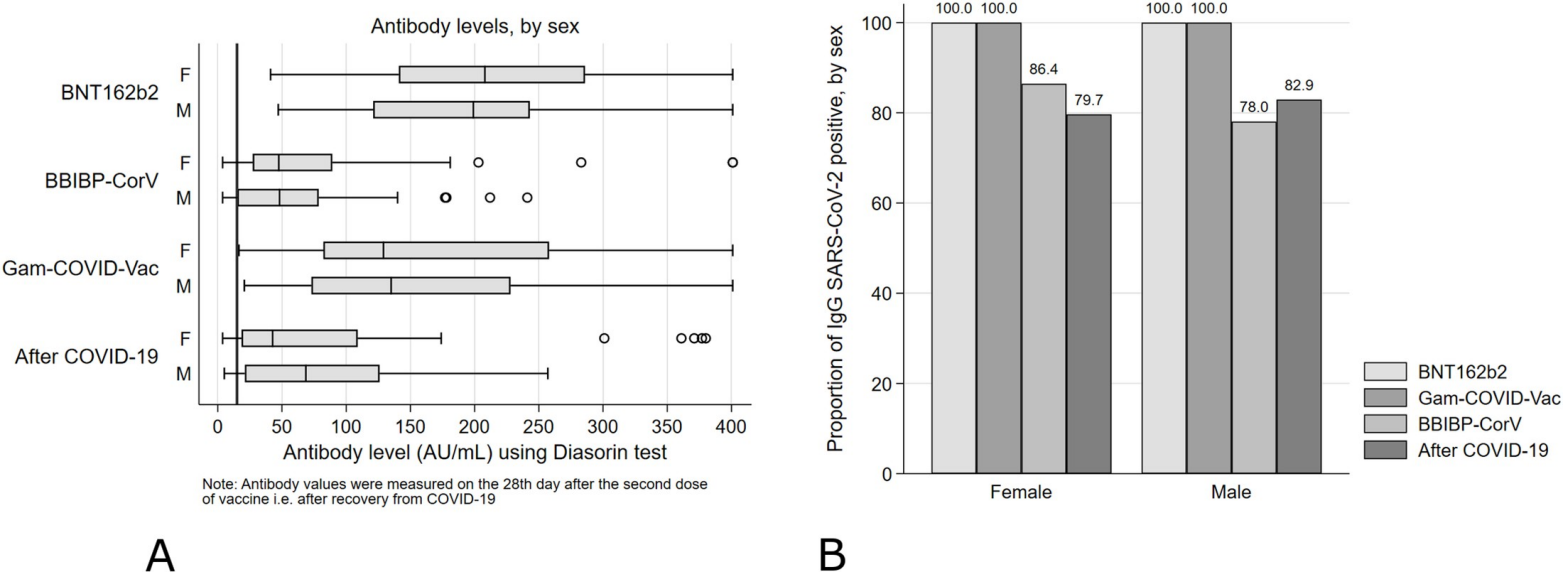
Antibody levels (A) and proportion of seropositive participants (B) by vaccine type and in COVID-19 convalescents according to sex of the participants. Thick vertical line on Fig 1(A) indicates the limit of reference level of protective antibodies (15.0 AU/mL).

The highest mean value of 210.11 (SD = 100.42) was found for the group vaccinated with BNT162b2, while in participants vaccinated with Gam-COVID-Vac and BBIBP-CorV mean values were 171.11 (SD = 120.69) and 68.50 (SD = 72.78) AU/mL, respectively (p<0.001). Similarly, comparison of antibody levels between three vaccinated groups showed that both in females and males, vaccinated with BNT162b2 had the highest values (mean = 215.85 and mean = 201.86) followed by those vaccinated with Gam-COVID-Vac (mean = 176.03 and mean = 164.03) and BBIBP-CorV (mean = 73.09 and mean 61.89), respectively (p<0.001, for both sexes). In all vaccine groups, females had slightly higher antibody levels than males, but the difference did not reach statistical significance in neither of them. The same pattern was noticed when stratifying the antibody levels by age, for each category from 30 to 69 years, where participants vaccinated with BNT162b2 reached the highest and BBIBP-CorV the lowest values in each age category (p<0.05, across four categories). On the other hand, regarding correlation between the antibody levels and the severity of COVID-19 disease in recovered participants, higher antibody levels were found in those who had more severe disease compared to those who had mild or asymptomatic infection: asymptomatic patients had mean level of 23.12 (SD = 27.30), mild 59.07 (SD = 50.04), severe 190.3 (SD = 120.9) while one participant who recovered from critical disease had antibody level of 142 AU/mL (p<0.001) (Table 1). A box-plot depicting antibody levels by sex, for three vaccine groups as well as after natural SARS-CoV-2 infection is presented in Fig 1A.

Interestingly, when comparing antibody levels between the vaccinated groups, we found a significant difference among those vaccinated with BNT162b2 and BBIBP-CorV (p<0.001), BNT162b2 and Gam-COVID-Vac (p = 0.001), as well as BBIBP-CorV and Gam-COVID-Vac (p<0.001) (S1–S3 Tables). Sex-based difference, both for males and females, was noticeable between vaccinated with BNT162b2 and BBIBP-CorV (p<0·001, for both sexes), BBIBP-CorV and Gam-COVID-Vac (p<0.001, for both sexes) while when comparing BNT162b2 and Gam-COVID-Vac, it reached statistical significance only for females (p = 0.011), with higher levels found in the BNT162b2 group. Regarding the age-based differences across the categories, participants vaccinated with BBIBP-CorV had siginificantly lower antibody values in comparison to BNT162b2 (age categories from 30 to 79 years, p<0.05 across the categories) and to Gam-COVID-Vac (age categories: 40–49, 50–59 and 70–79, p<0.05 across the categories).

Detailed data on antibody levels for each of the three analyzed vaccine types compared to those of COVID-19 recovered individuals and stratified by sex, age, and clinical presentation of COVID-19 are presented in S4–S6 Tables. Significantly higher antibody levels were found in individuals vaccinated with BNT162b2 (p<0.001) and Gam-COVID-Vac (p<0.001) when compared to levels in COVID-19 recovered patients, while for those vaccinated with BBIBP-CorV there was no statistically significant difference between vaccinated and convalescent group (p = 0.641). The same pattern was observed when antibody levels among males and females were compared between COVID-19 convalescents and each of the vaccinated groups. Regarding differences across age categories, younger categories of participants vaccinated with BNT162b2 and Gam-COVID-Vac (age 30–59) had higher antibody values in respect to those recovered from COVID-19 (p<0.001, for age categories).

**Fig 2:**
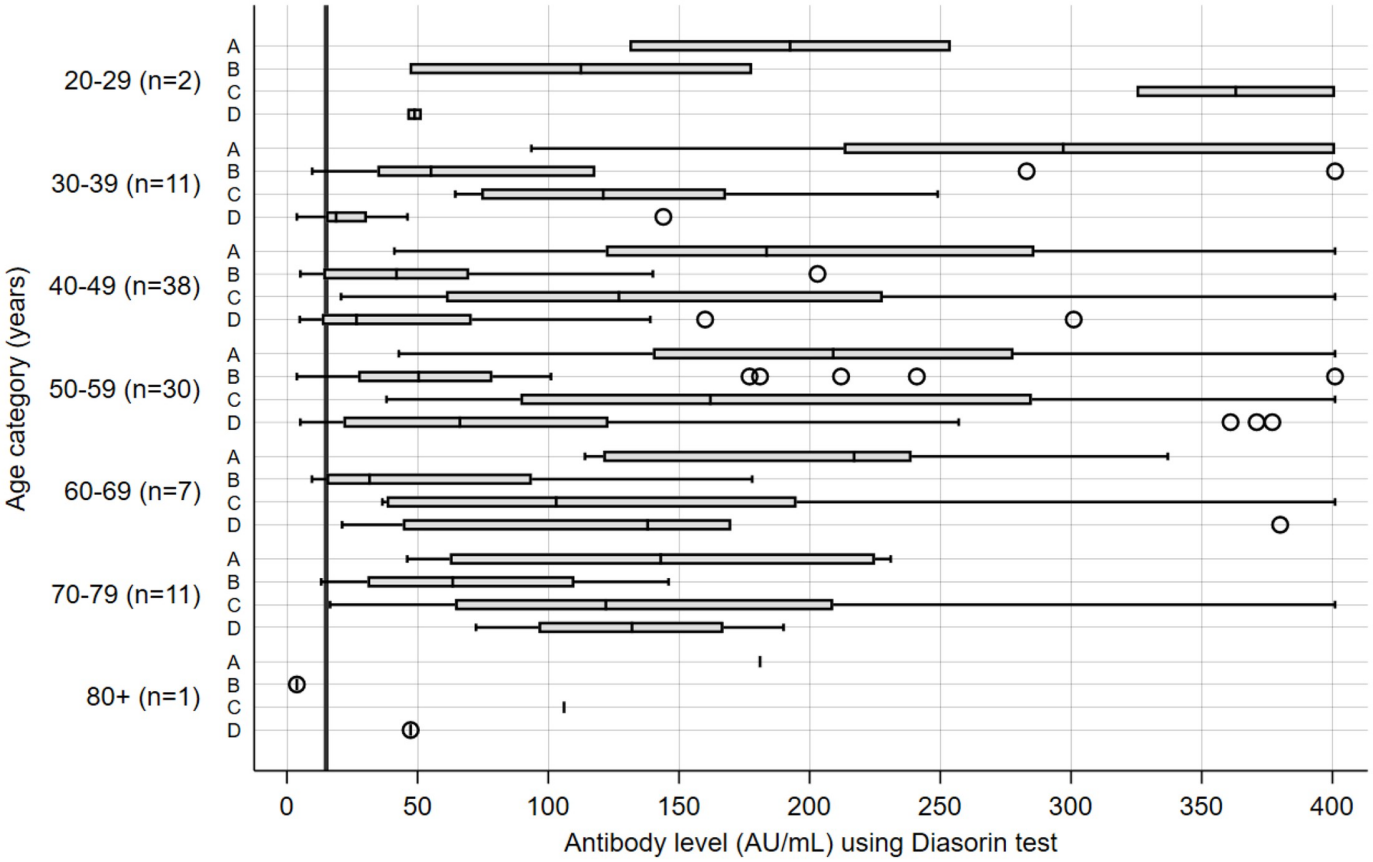
Antibody levels after BNT162b2 (A), BBIBP-CorV (B) and Gam-COVID-Vac (C) vaccine and after recovery from COVID-19 (D) stratified by different age-categories. Thick vertical line indicates the limit of reference level of protective antibodies (15.0 AU/mL). n = number of participants in each of the groups (A, B, C, D) within the age-category. Note: Antibody values were measured on the 28^th^ day after the second dose of vaccine or after recovery from COVID-19.

Further, we analyzed the seroconversion rate in vaccinated persons and in convalescents. The percentage of vaccinated participants that resulted seropositive ranged from 83% after BBIBP-CorV to 100% after BNT162b2 and Gam-COVID-Vac, while in the COVID-19 recovered group 81% of participants were seropositive. When stratifying seropositivity to SARS-CoV-2 by sex, we noticed that 86.4% of females and 78.1% of males were seropositive after the BBIBP-CorV (p = 0.221) while 79.7% of females and 82.9% males were seropositive after COVID-19 (p = 0.92) (Fig 1B). We also compared seropositivity rates and antibody levels in different vaccinated groups and in convalescents, stratified by different age categories. Lowest percent of seropositivity, apart from one single seronegative participant in the oldest age category (80+) vaccinated with BBIBP-CorV, was observed in participants in age category 40–49, both after BBIBP-CorV vaccine and after COVID-19 (71.1%) (S1 Fig). Finally, analyses of antibody levels stratified by 10-year age categories revealed generally higher levels in younger vaccinated population, after all three types of vaccines, while in the group of convalescent participants, the highest antibody levels were observed in older age categories, as shown in Fig 2.

**Fig 3:**
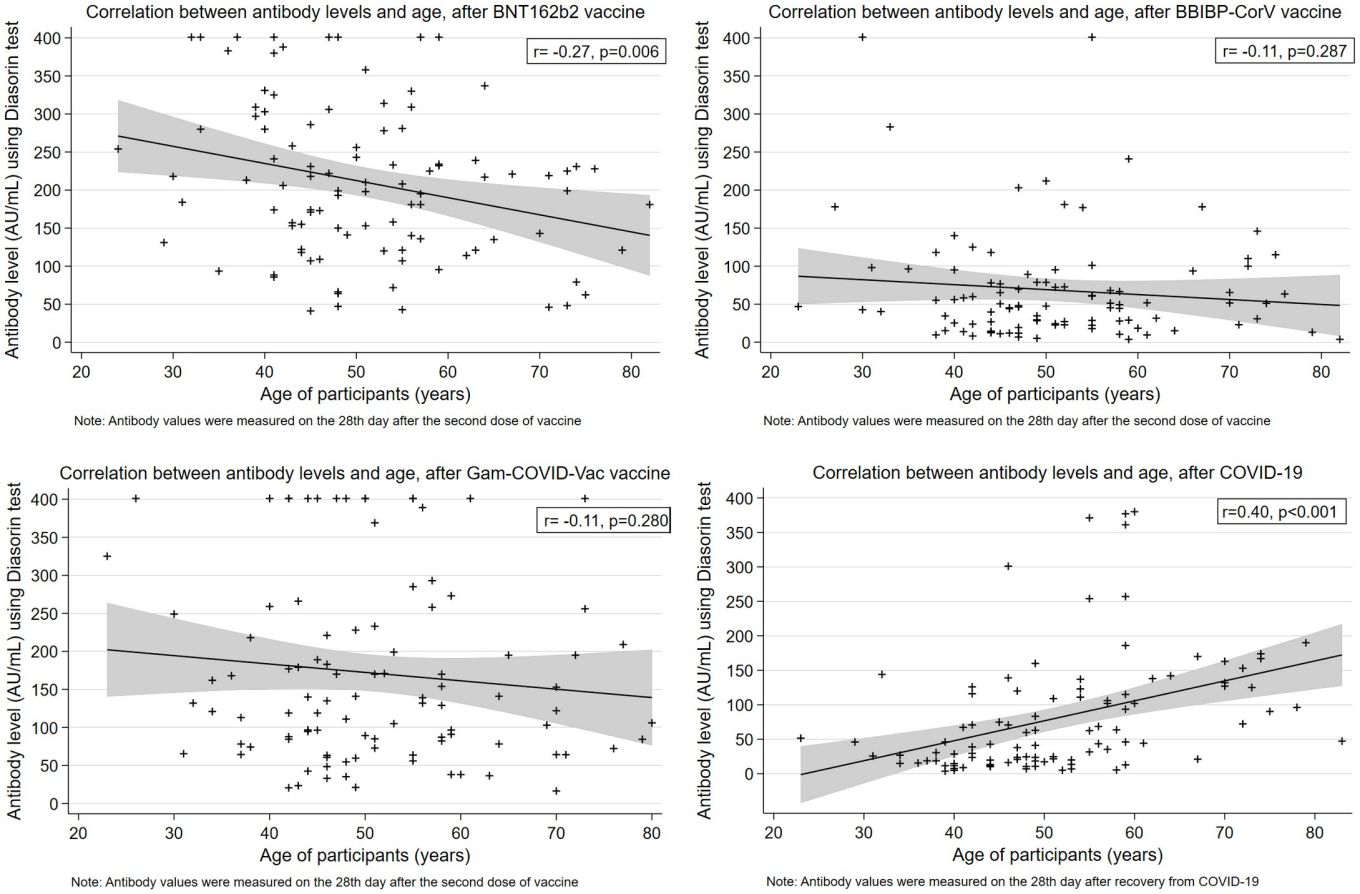
Correlation between antibody levels and age, by vaccine type and after COVID-19 recovery.

In addition, we found a significant weak negative correlation between antibody levels and age of participants for the group vaccinated with BNT162b2 (r = -0.27, p = 0.006), whereas similar trend was also observed for the other two vaccines, but it was not statistically significant (Fig 3).

On the other hand, a significant moderate positive correlation between antibody levels and age was observed in participants recovered from a natural SARS-CoV-2 infection (r = 0.40, p<0.001) (Fig 3). Finally, we noticed a moderate positive correlation between antibody levels after recovery from COVID-19 and the severity of clinical presentation of the disease (r = 0.57, p<0.001) (S2 Fig).

## Discussion

Measuring levels of serum antibodies allows comparison of immunogenicity between different groups of vaccinated and immunity in convalescent patients. To the best of our knowledge, this is the first study to evaluate magnitude of the antibody response after three available COVID-19 vaccines and after a natural SARS-CoV-2 infection, in such a comparative term. Our findings regarding the specific vaccine types demonstrate that the highest mean value was measured in the group vaccinated with BNT162b2, followed by Gam-COVID-Vac and BBIBP-CorV, while the antibody levels did not significantly differ in males and females, in neither of the vaccine groups. In general, higher levels of antibodies were measured in younger population of vaccinated after all three types of vaccines in contrast to the convalescents after natural COVID-19 infection where participants from older categories had higher antibody levels in respect to younger categories. All individuals vaccinated with BNT162b2 and Gam-COVID-Vac seroconverted four weeks after the second dose, while percentage of seropositive was lower in those vaccinated with BBIBP-CorV and in the group of COVID-19 recovered individuals (83% and 81%, respectively).

The SARS-CoV-2 has four structural proteins, spike surface glycoprotein (S), membrane, envelope and nucleocapsid proteins thatare crucial in the process of infection [14]. Its trimeric spike protein is used in a multi-step process of binding to the host’s cells angiotensin-converting enzyme 2 (ACE2) receptors and fusing with the membrane to enter the cell [15]. The S protein is also a major immunogen and the target of Ab-mediated neutralization during a response to coronavirus infections. It consists of S1 and S2 subunits, where S1 contains the receptor-binding domain (RBD) that binds to cell’s ACE2 receptor, while S2 is responsible for the membrane fusion [16]. Additionally, many of the current SARS-CoV-2 vaccines aim to induce synthesis of the neutralizing anti-S antibodies after vaccine administration [3]. The levels of neutralizing antibodies are usually measured using neutralization assays, which detect only functional antibodies capable of blocking virus entry into the target cells. These tests are not standardized, they are complicated to perform and come in many variations with pseudo-virus or replicating virus. Other assays, such as the enzyme-linked immunosorbent assays (ELISAs) or CLIA method, are easier to perform, but they measure total amount of antibodies that bind to virus proteins (mostly S protein) including neutralizing antibodies. In general, neutralizing antibody levels, as measured by neutralization assays, correlate reasonably well with levels of antibodies binding to S protein that are measured by such “binding” assays [4]. We used an assay which is able to detect and quantify IgG antibodies against S1/S2 subunits with a high sensitivity and specificity using the CLIA method and which has been shown to have 94.4% positive agreement to Plaque Reduction Neutralization Test [13].

When comparing antibody levels between the groups vaccinated with different vaccine platforms, we noticed the highest values in those vaccinated with BNT162b2, followed by Gam-COVID-Vac and BBIBP-CorV. Similar differences have been previously reported also for the estimated efficacies of these three vaccines, namely 95% for BNT162b2, 91.4% for Gam-COVID-Vac and 72.8% for the BBIBP-CorV [4, 17], even though the assesment of the endpoints varied between different trials. The observed difference in the immunogenicity might be due to diverse platforms used for development of specific vaccines. Although we demonstrated lower immunogenicity of BBIBP-CorV in respect to other two vaccines, it is possible that BBIBP-CorV may induce broader immune response that include antibodies other than those against spike protein, which could be of particular importance for protection against virus variants with mutations in the spike region [4, 18]. In our study, both BNT162b2 and Gam-COVID-Vac vaccines demonstrated stronger initial peak response in comparison to BBIBP-CorV, and it is reasonable to expect that the Ab levels after these two vaccines will drop at a slower rate than after BBIBP-CorV [4]. Therefore, studies on vaccinated individuals at later time-points, such as 6 and 12 months after the second dose, are warranted to further explore this.

Regarding the percentage of seropositives, it reached 100% after BNT162b2 and Gam-COVID-Vac, while after BBIBP-CorV vaccine it was 83%. For the BNT162b2, our results are similar to high immunogenicity demonstrated in initial trials [19] and some similar studies [2]. In phase 1/2 trial of the BBIBP-CorV, a robust humoral response with 100% seroconversion was found in all participants on day 42 after the second dose [8]. Similarly, in phase 3 randomized trial conducted in the Middle East, the seroconversion rate was 99.3%, measured 14 days after two doses of immunization [17]. These findings were not confirmed by our study on the 28^th^ day after the second dose of BBIBP-CorV. On the other hand, in the group of COVID-19 recovered, 81% of participants were seropositive, higher than in the study by Zhao et al. which demonstrated a seroconversion rate of 64.7% for IgG antibodies on 173 SARS-CoV-2 postitive confirmed patients [20].

Higher levels of antibody were mostly found in younger population in the vaccinated groups of all three types of vaccines, and in older age categories after natural COVID-19 infection. A study by Röltgen and coworkers also demonstrated higher antibody levels in younger participants compared to those over 60 years, in a cohort of 55 BNT162b2 vaccine recipients, measured at days 28 and 42 after their first vaccination (preprint data, not yet peer reviewed) [21]. Immunosenescence, a declining immunity with age is a known phenomenon that affects both cell-mediated and humoral immunity, with a decreasing number of naive T cells, T cell exhaustion, as well as production of fewer functional antibodies by B-cells, which could be a basis for lower immunogenicity and effectiveness of vaccines generally observed in elderly [22]. In addition to the quantitative loss of naïve T cells, immune response following vaccination in aged persons inclines towards the short-lived effector T-cells over the long-term memory precursors and follicular helper T (T_FH_) cells that mediate high-affinity Ab production leading to a lower protective capacity of vaccine-induced antibodies [22]. Similarly, B cells also demonstrate impaired function with age, with the stimulation of the proinflammatory subsets possibly driven by the local tissue inflammation [26]. In fact, persistent low-grade inflammation and higher titres of pro-inflammatory molecules (termed inflammaging), commonly seen in the elderly, can hinder B-cell response to stimulation, and consequently reduce the Ab production [23, 24]. As a result, this population is generally under increased risk of serious COVID-19 and poorer outcome, thus a particular attention should be placed to further investigate vaccine effectiveness among them. On the other hand, higher levels of antibodies in older COVID-19 convalescent patients in our study seem to be in contradiction with lower response to vaccines in these age categories. A cross-sectional study conducted in the New York City hospital compared IgG levels between 85 pediatric and 3648 adult COVID-19 patients and showed a moderate positive correlation of SARS-CoV-2 IgG levels with age in adults (*r* = 0.24, *P* < 0.001) [25], which is in line with our finding in the group of participants recovered from a natural SARS-CoV-2 infection. Similarly, Luo et al. concluded that advancing age is associated with higher antibody response and hyperinflammation induced by multiple chemokines in COVID-19 patients [12]. However, other study, in a smaller cohort of patients, within the same disease severity group, found no correlation betwen the age and the magnitude of humoral response and suggested that, in fact, the severity of disease is guiding this correlation [26] which may also be the case in our cohort, since higher percentage of severe cases of COVID-19 was present in older in respect to younger categories. Yet, these findings should be further confirmed in studies that will include much more participants from older categories with severe (and critical) clinical presentation of COVID-19.

We also demonstrated that the mean antibody concentrations were higher in the vaccinated group with BNT162b2 and Gam-COVID-Vac than in the COVID-19 recovered participants. This result is in line with the findings from previous studies. A recent study showed that vaccination with BNT162b2 induced highly targeted IgG anti-S Abs whose levels were higher than those in mildly or moderately ill COVID-19 patients but similar to those who were severely ill, at days 28 and 42 (preprint data, not yet peer reviewed) [21]. Other studies demonstrated a peak in antibody responses ranging from half to four times higher in those vaccinated when compared to those in convalescent patients [27, 28]. Question that remains open is the durability of these immune responses over time, but some early findings suggest that decline rate in antibody responses in the first months is not significantly different in vaccinated and those recovered from COVID-19 infection [27].

Our study has several limitations that should be addressed. Although our sample size was relatively small across the investigated groups, we used rigorous inclusion criteria with age- and sex-matched participants to make robust comparison. Also, we included participants from wide age range, and for the group with COVID-19 convalescents we covered different levels of disease severity—from asymptomatic to critical. Next, we lack information on co-morbidities and chronic therapies of the participants that may have affected their immune response to vaccines or SARS-CoV-2 infection. Further, we did not measure levels of Abs before vaccine administration, and even though we included only vaccinated individuals who had not been previously tested positive for SARS-CoV-2 (either by PCR or RDT-Ag), we cannot exclude the possibility that some of them contracted the virus in the past and/or had a previous asymptomatic infection, which may have also influenced the Ab levels measured in their sera. Although the measurement of Ab levels against N-protein could help to identify those exposed to a virus prior to vaccination, but asymptomatic, especially among those vaccinated with vaccines containing only S protein (BNT162b2 and Gam-COVID-Vac in our study), the assessment of anti-N antibodies was not available to us. The correlates of protection against SARS-CoV-2 have not been defined yet, but it has been clearly shown that higher anti-spike IgG, anti-RBD IgG, and neutralizing antibody titers are all associated with lower risk of symptomatic disease [29] and anti-S antibodies are considered to be a great humoral immune marker used to estimate post-infection or post-vaccination immunity. Nevertheless, measuring IgG antibody to spike protein might not be enough to determine complete immunity with regard to vaccine effectiveness, in particular in individuals vaccinated with BBIBP-CorV, and those recovered from natural infection, since IgG to other SARS-CoV-2 antigens are also produced. Independent from the humoral response, SARS-CoV-2 infection also produces a cellular response by activating a variety of T cells against all major SARS-CoV-2 antigens. Even if no antibodies are detectable, a strong post-infection cellular response would provide long-term protection against SARS-CoV-2 infection [10, 30, 31], as it has already been shown for some other infections including MERS [32]. Therefore, it would be of interest to assess the T cell response in addition to B cell response, but this was beyond the scope of this study. However, since antibody levels are measured after a short period of time (28 days) after vaccination or infection, we may consider that the measured antibody levels in that time period would correlate well with the level of individual protection from infection recurrence.

In conclusion, our results provide evidence that vaccination, with all three investigated vaccines namely BNT162b2, BBIBP-CorV and Gam-COVID-Vac provide robust immune response 28 days after the second dose of vaccine, in the majority of participants. All individuals vaccinated with BNT162b2 and Gam-COVID-Vac seroconverted, while in those vaccinated with BBIBP-CorV, the percentage of seropositive participants was lower. BBIBP-CorV was also less potent in anti-S antibody induction compared to other two vaccines. However, BBIBP-CorV induced similar levels of anti-S antibodies to those measured in convalescents implying therefore that its protective capacity against disease-related complications and severe outcomes of COVID-19 may be expected to be satisfactory, although responses to other SARS-CoV-2 antigens have not been evaluated in our study. We believe that our results are of particular importance since this is one of the few studies that present Ab responses in comparative terms, between different vaccine platforms in a similar population. As the immunization continues to progress worldwide, major challenge remains to examine dynamics and durability of immunoprotection, and further follow-up studies over months (and years) following vaccination and recovery from COVID-19 are warranted to investigate this. Also, large-scale cross-national population studies are needed to further determine vaccine effectiveness after their implementation in the national immunization programme, in particular after the emergence of new virus variants. Our results may further help in planning the third dose of vaccine (to estimate the necessity of a booster-dose) and also provide evidence for modifying current recommendations for vaccination in persons that have recovered from SARS-CoV-2 infection. Finally, our findings strongly support the benefits of vaccination against SARS-CoV-2 as a preventive measure.

## Supporting information

S1 Fig Proportion of IgG SARS-CoV-2 seropositive participants by 10-year age categories. (TIF)

S2 Fig Correlation between antibody levels after recovery from COVID-19 and severity of clinical presentation of COVID-19. (TIF)

S1 Table Antibody levels on the 28^th^ day from the administration of the second dose of the BNT162b2 and BBIBP-CorV vaccine, stratified by sex and age. (DOCX)

S2 Table Antibody levels on the 28^th^ day from the administration of the second dose of the BNT162b2 and Gam-COVID-Vac vaccine, stratified by sex and age. (DOCX)

S3 Table Antibody levels on the 28^th^ day from the administration of the second dose of the BBIBP-CorV and Gam-COVID-Vac vaccine, stratified by sex and age. (DOCX)

S4 Table Antibody levels on the 28^th^ day from the administration of the second dose of the BNT162b2 vaccine or after COVID-19 recovery, stratified by sex and age. (DOCX)

S5 Table Antibody levels on the 28^th^ day from the administration of the second dose of the BBIBP-CorV vaccine or after COVID-19 recovery, stratified by sex and age. (DOCX)

S6 Table Antibody levels on the 28^th^ day from the administration of the second dose of the Gam-COVID-Vac vaccine or after COVID-19 recovery, stratified by sex and age. (DOCX)
