# Supplementary figures and images for: Immunogenicity of BNT162b2, BBIBP-CorV and Gam-COVID-Vac vaccines and immunity after natural SARS-CoV-2 infection—A comparative study from Novi Sad, Serbia
Source: PLoS One. 2022 Feb 2;17(2):e0263468. doi: 10.1371/journal.pone.0263468 (PMC8809561; doi:10.1371/journal.pone.0263468)

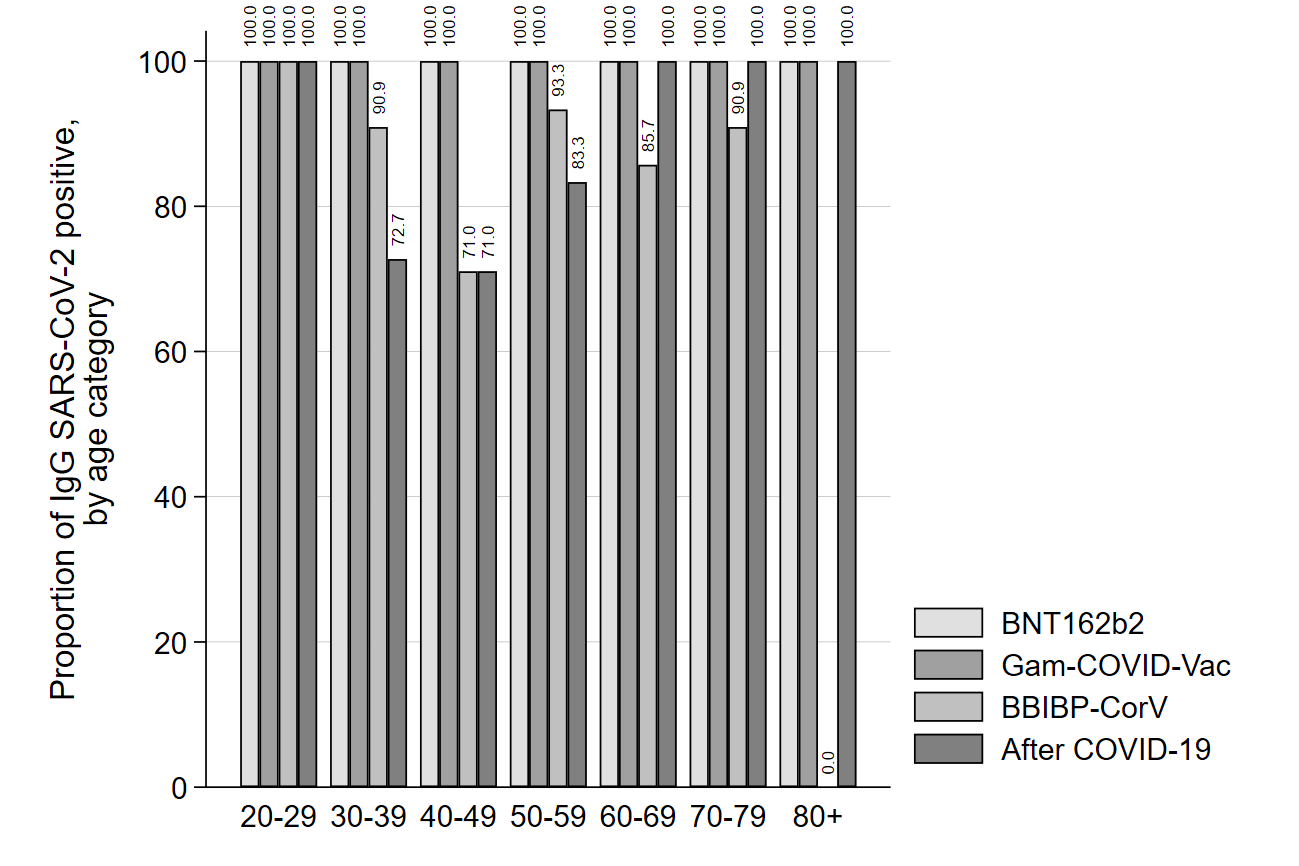

Supplement: S1 Fig — (TIF) [file pone.0263468.s001.tif]

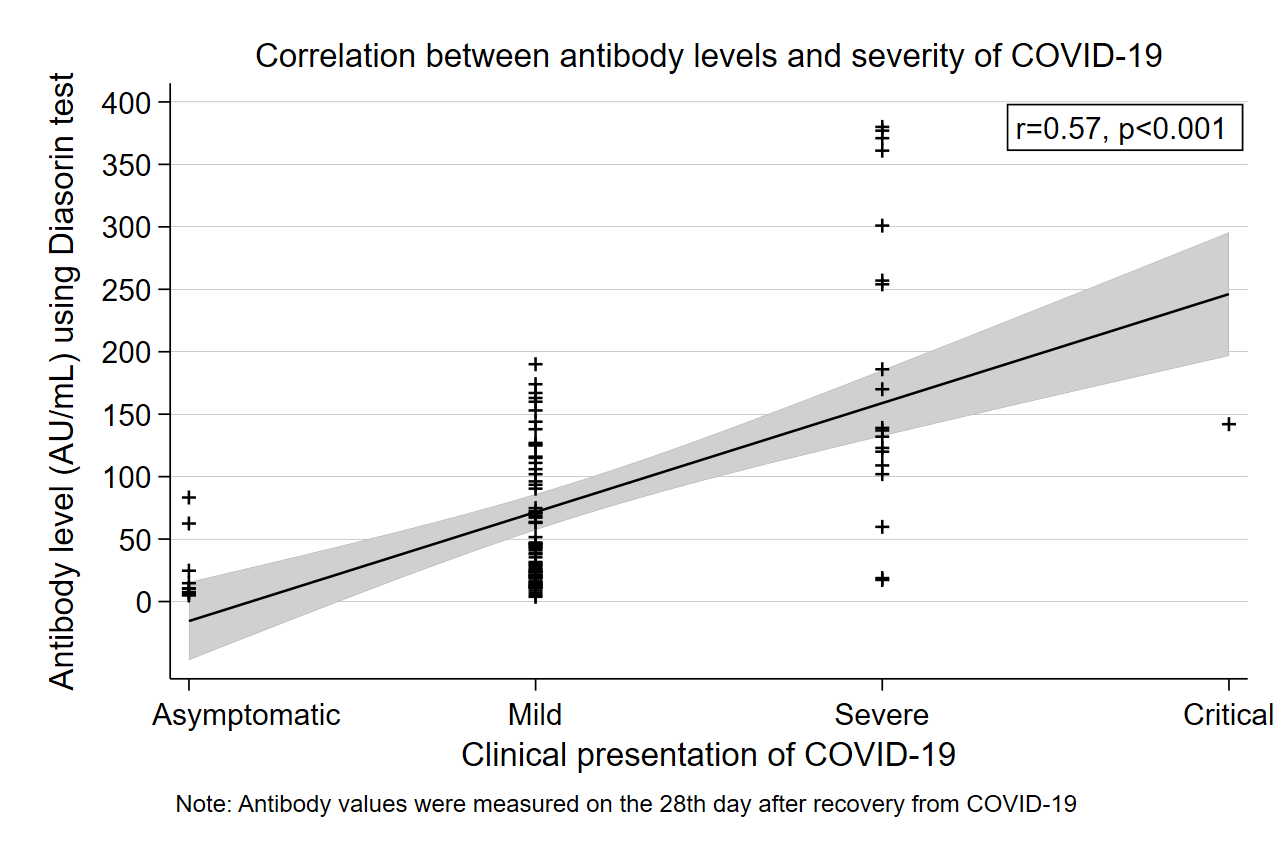

Supplement: S2 Fig — (TIF) [file pone.0263468.s002.tif]
