# Supplementary material for: Immunogenicity of BNT162b2, BBIBP-CorV and Gam-COVID-Vac vaccines and immunity after natural SARS-CoV-2 infection—A comparative study from Novi Sad, Serbia
Source: PLoS One. 2022 Feb 2;17(2):e0263468. doi: 10.1371/journal.pone.0263468 (PMC8809561; doi:10.1371/journal.pone.0263468)
Supplement: S2 Table — (DOCX) [file pone.0263468.s004.docx]

**S2 Table. Antibody levels on the 28^th^ day from the administration of the second dose of the BNT162b2 and Gam-COVID-Vac vaccine, stratified by sex and age.**

|  | **Participants (%)** | **BNT162b2 vaccine (n=100)** | | | | | **Gam-COVID-Vac vaccine (n=100)** | | | | |  |
| --- | --- | --- | --- | --- | --- | --- | --- | --- | --- | --- | --- | --- |
|  |  | **mean (AU/mL)** | **SD** | **median (AU/mL)** | **IQR (25-75)** | | **mean (AU/mL)** | **SD** | **median (AU/mL)** | **IQR (25-75)** | | **p-value^1^** |
| **Total** | 100 | 210.11 | 100.42 | 207.00 | 133.00 | 280.00 | 171.11 | 120.69 | 133.5 | 78.25 | 241 | 0.001 |
| **Sex** |  | | | | | |  | | | | |  |
| Male | 41 | 201.86 | 102.12 | 199.00 | 121.00 | 243.00 | 164.03 | 114.31 | 135.00 | 72.90 | 228.00 | 0.057 |
| Female | 59 | 215.85 | 99.69 | 208.00 | 141.00 | 286.00 | 176.03 | 125.66 | 129.00 | 82.20 | 258.00 | 0.011 |
| **Age category** |  | | | | | |  | | | | |  |
| 20-29 | 2 | 192.50 | 86.97 | 192.50 | 131.00 | 254.00 | 363.00 | 53.74 | 363.00 | 325.00 | 401.00 | 0.333 |
| 30-39 | 11 | 289.14 | 103.48 | 297.00 | 213.00 | 401.00 | 131.40 | 62.43 | 121.00 | 74.30 | 168.00 | 0.001 |
| 40-49 | 38 | 207.01 | 106.25 | 183.50 | 122.00 | 286.00 | 167.57 | 129.63 | 127.00 | 60.80 | 228.00 | 0.061 |
| 50-59 | 30 | 213.77 | 92.72 | 209.00 | 140.00 | 278.00 | 194.50 | 122.35 | 162.00 | 89.30 | 285.00 | 0.268 |
| 60-69 | 7 | 197.71 | 80.46 | 217.00 | 121.00 | 239.00 | 141.84 | 127.41 | 103.00 | 38.10 | 195.00 | 0.165 |
| 70-79 | 11 | 145.59 | 77.48 | 143.00 | 62.30 | 225.00 | 148.89 | 111.07 | 122.00 | 64.30 | 209.00 | 0.949 |
| 80+ | 1 | 181.00 | NA | 181.00 | NA | NA | 106.00 | NA | 106.00 | NA | NA | 1.000 |

Note: For statistical processing and presentation of data, results below the minimum detectable value of the assay (<3.8) were interpreted as 3.79, and above the maximum detectable value (> 400) as 401. ^1^Wilcoxon rank-sum (Fisher’s exact test where appropriate); p-value refers to difference between variables within the same group. NA=not applicable. n=number of participants in each study group.
