# Supplementary material for: Immunogenicity of BNT162b2, BBIBP-CorV and Gam-COVID-Vac vaccines and immunity after natural SARS-CoV-2 infection—A comparative study from Novi Sad, Serbia
Source: PLoS One. 2022 Feb 2;17(2):e0263468. doi: 10.1371/journal.pone.0263468 (PMC8809561; doi:10.1371/journal.pone.0263468)
Supplement: S3 Table — (DOCX) [file pone.0263468.s005.docx]

**S3 Table. Antibody levels on the 28^th^ day from the administration of the second dose of the BBIBP-CorV and Gam-COVID-Vac vaccine, stratified by sex and age.**

|  | **Participants (%)** | **BBIBP-CorV vaccine (n=100)** | | | | | **Gam-COVID-Vac vaccine (n=100)** | | | | |  |
| --- | --- | --- | --- | --- | --- | --- | --- | --- | --- | --- | --- | --- |
|  |  | **mean (AU/mL)** | **SD** | **median (AU/mL)** | **IQR (25-75)** | | **mean (AU/mL)** | **SD** | **median (AU/mL)** | **IQR (25-75)** | | **p-value^1^** |
| **Total** | 100 | 68.50 | 72.78 | 47.80 | 22.95 | 83.95 | 171.11 | 120.69 | 133.5 | 78.25 | 241 | <0.001 |
| **Sex** |  | | | | | |  | | | | |  |
| Male | 41 | 61.89 | 58.38 | 48.10 | 15.20 | 78.60 | 164.03 | 114.31 | 135.00 | 72.90 | 228.00 | <0.001 |
| Female | 59 | 73.09 | 81.48 | 47.50 | 27.20 | 89.20 | 176.03 | 125.66 | 129.00 | 82.20 | 258.00 | <0.001 |
| **Age category** |  | | | | | |  | | | | |  |
| 20-29 | 2 | 112.45 | 92.70 | 112.45 | 46.90 | 178.00 | 363.00 | 53.74 | 363.00 | 325.00 | 401.00 | 0.333 |
| 30-39 | 11 | 108.54 | 123.34 | 55.10 | 34.60 | 118.00 | 131.40 | 62.43 | 121.00 | 74.30 | 168.00 | 0.101 |
| 40-49 | 38 | 49.91 | 43.27 | 41.90 | 13.90 | 69.70 | 167.57 | 129.63 | 127.00 | 60.80 | 228.00 | <0.001 |
| 50-59 | 30 | 78.77 | 85.36 | 50.35 | 27.20 | 78.60 | 194.50 | 122.35 | 162.00 | 89.30 | 285.00 | <0.001 |
| 60-69 | 7 | 56.88 | 60.77 | 31.60 | 15.10 | 93.70 | 141.84 | 127.41 | 103.00 | 38.10 | 195.00 | 0.073 |
| 70-79 | 11 | 69.95 | 42.48 | 63.40 | 30.80 | 110.00 | 148.89 | 111.07 | 122.00 | 64.30 | 209.00 | 0.040 |
| 80+ | 1 | 3.79 | NA | 3.79 | NA | NA | 106.00 | NA | 106.00 | NA | NA | 1.000 |

Note: For statistical processing and presentation of data, results below the minimum detectable value of the assay (<3.8) were interpreted as 3.79, and above the maximum detectable value (> 400) as 401. ^1^Wilcoxon rank-sum (Fisher’s exact test where appropriate); p-value refers to difference between variables within the same group. NA=not applicable. n=number of participants in each study group
