# Supplementary material for: Immunogenicity of BNT162b2, BBIBP-CorV and Gam-COVID-Vac vaccines and immunity after natural SARS-CoV-2 infection—A comparative study from Novi Sad, Serbia
Source: PLoS One. 2022 Feb 2;17(2):e0263468. doi: 10.1371/journal.pone.0263468 (PMC8809561; doi:10.1371/journal.pone.0263468)
Supplement: S5 Table — (DOCX) [file pone.0263468.s007.docx]

**S5 Table. Antibody levels on the 28^th^ day from the administration of the second dose of the BBIBP-CorV vaccine or after COVID-19 recovery, stratified by sex and age.**

|  | **Participants (%)** | **BBIBP-CorV vaccine (n=100)** | | | | | **COVID-19 recovered (n=100)** | | | | |  |
| --- | --- | --- | --- | --- | --- | --- | --- | --- | --- | --- | --- | --- |
|  |  | **mean (AU/mL)** | **SD** | **median (AU/mL)** | **IQR (25-75)** | | **mean (AU/mL)** | **SD** | **median (AU/mL)** | **IQR (25-75)** | | **p-value^1^** |
| **Total** | 100 | 68.50 | 72.78 | 47.80 | 22.95 | 83.95 | 81.23 | 86.46 | 46.10 | 19.30 | 121.50 | 0.641 |
| **Sex** |  | | | | | |  | | | | |  |
| Male | 41 | 61.89 | 58.38 | 48.10 | 15.20 | 78.60 | 83.12 | 67.81 | 68.60 | 21.10 | 126.00 | 0.148 |
| Female | 59 | 73.09 | 81.48 | 47.50 | 27.20 | 89.20 | 79.92 | 97.90 | 42.70 | 18.50 | 109.00 | 0.498 |
| **Age category** |  | | | | | |  | | | | |  |
| 20-29 | 2 | 112.45 | 92.70 | 112.45 | 46.90 | 178.00 | 48.80 | 3.96 | 48.80 | 46.00 | 51.60 | 0.667 |
| 30-39 | 11 | 108.54 | 123.34 | 55.10 | 34.60 | 118.00 | 32.42 | 38.65 | 18.80 | 14.90 | 30.60 | 0.034 |
| 40-49 | 38 | 49.91 | 43.27 | 41.90 | 13.90 | 69.70 | 51.39 | 58.49 | 26.60 | 13.30 | 70.70 | 0.540 |
| 50-59 | 30 | 78.77 | 85.36 | 50.35 | 27.20 | 78.60 | 106.06 | 111.53 | 66.15 | 21.60 | 123.00 | 0.523 |
| 60-69 | 7 | 56.88 | 60.77 | 31.60 | 15.10 | 93.70 | 142.49 | 117.76 | 138.00 | 44.30 | 170.00 | 0.097 |
| 70-79 | 11 | 69.95 | 42.48 | 63.40 | 30.80 | 110.00 | 135.45 | 37.78 | 132.00 | 96.20 | 167.00 | 0.002 |
| 80+ | 1 | 3.79 | NA | 3.79 | NA | NA | 47.30 | NA | 47.30 | NA | NA | 1.000 |

Note: For statistical processing and presentation of data, results below the minimum detectable value of the assay (<3.8) were interpreted as 3.79, and above the maximum detectable value (> 400) as 401. ^1^Wilcoxon rank-sum (Fisher’s exact test where appropriate); p-value refers to difference between variables within the same group. NA=not applicable. n=number of participants in each study group
